# American Ass’n of Obstetricians and Gynecologists

**Published:** 1909-11

**Authors:** 


### American Ass'n of Obstetricians and Gynecologists

THIS active body of specialists held its twenty-second annual meeting at Fort Wayne, Indiana, Tuesday, Wednesday and Thursday, September 21, 22 and 23, 1909, under the presidency of Dr. William H. Humiston, of Cleveland. This is probably the first instance in which a national special medical society has held an annual meeting in one of the smaller cities of the country. It is customary to hold such meetings in the medical centers, where the population is large, and the number interested is many. The meeting in question, however, proved the wisdom of selecting Fort Wayne, and disproved the custom of adhering to populous cities in fixing places for holding these meetings.

It is, in fact, the smaller cities that manifest the largest interest in medical gatherings of this kind. The medical profession of Fort Wayne and vicinity, turned out in large numbers to the several sessions, coming early and staying late, so great was the interest manifested.

The Chairman of the Committee of Arrangements, Dr. Miles F. Porter, conducted the local affairs of the association with great discretion, and created an interest in advance of the meeting that held all through its several sessions.

The Hotel Anthony in which the meeting was held is a new structure, built on the most approved plan, and contains ample and delightful accommodations for a convention like this one. Mr. Reagan, the proprietor, understands the art of entertainment, and of making his guests feel at home.

The banquet on Wednesday night was all that could be desired in the way of delightful social entertainment. The music was good and the speeches crisp. The association chose for the place of its next meeting, the city of Syracuse in the state of New York. This is also a departure from the usual custom, but it will be remembered that Syracuse is in the center of a very populous region, one central to education and refinement, and is accessible by rapidly running trains to very many of the largest cities in the union.

The clinic held at one of the local hospitals by Dr. John Young Brown, of Saint Louis, on the morning of the third day was instructive, and again demonstrated the ability of that brilliant operator to deal with difficult surgical problems.

Dr. Aaron B. Miller, president-elect, presented the claims of Syracuse, his own city, with such cogent force and in such a winning manner that the association accepted promptly and unanimously. The time fixed for the meeting was September 20, 21, and 22, 1910, and the profession of medicine is cordially invited to attend and participate in the proceedings.

The officers elected for the ensuing year are: president, Aaron B. Miller, Syracuse, N. Y.; vice-president, Charles North Smith, Toledo, Ohio; vice-president, Raleigh Russell Huggins, Pittsburg, Pa.; secretary, William Warren Potter, Buffalo; treasurer, Xavier Oswald Werder, Pittsburg, Pa.; executive counselors, William H. Humiston, Cleveland, Ohio; Hugo Otto Pantzer, Indianapolis, Ind.

---

THE Queen Alexandra Sanatorium, at Davos, which is noticed elsewhere is unique in its purposes, in receiving patients from all parts of the world. The prospective opening of the sanatorium for the reception of patients early in this autumn was announced from the chair at the sixth annual meeting of the Council, held at 11 Chandos street, Cavendish Square, W., on July 16, by the President, the Lord Balfour of Burleigh, K.T., P.C., who has labored so long and successfully in the difficult task of raising funds. A splendid donation of £25,000 lately received from a munificent sympathiser, who desires that his name shall not be published, not only supplies the amount required to complete the work and to open the sanatorium free from debt, but provides means for its scientific equipment and for future extensions. It should be mentioned that Lord Strathcona, with his well-known zeal in the promotion of all charitable and useful works, not long ago gave a donation of £2,000 for the purposes of the sanatorium. For the present the sanatorium will accommodate 54 patients, all in single rooms. But the public rooms are designed for a full complement of 120 patients. The Davos Invalids' Home, the original foundation of the late Mrs. Lord, which for so many years was the only representative of our national charity in Davos, has now ended its task and fulfilled the purpose for which it was initiated—that of developing into a National Sanatorium. The Home had been granted Her Gracious Majesty's patronage as far back as 1899.

CALOX, the oxygen tooth powder (McKesson & Robbins), represents not only cleanliness for the mouth and teeth, but treatment for the gums and other soft parts. It is the tooth powder of refinement and intelligence; it not only gives freshness to the entire mouth, but sweetness to the breath, as well as a pure white cleanliness to the teeth. It is *the* oxygen tooth powder.

---

THE New Board of Examiners in Midwifery, for the County of Erie, is composed of the following-named members: For one year—Nahum Kavinoky, Lesser Kauffman, Frederick J. Parmenter; for two years—Charles S. Jewett, Guiseppe Tartaro, William H. Thornton; for three years—John A. Pettit, Ludwig Schroeter, Peter W. VanPeyma. The appointments were made by Honorable Harry L. Taylor, County Judge, who is vested by law with this authority, and he has chosen an excellent board.

---

AT the invitation of the trustees of Roosevelt Hospital, the surgeons of the German, French, English and Italian visiting warships attended a surgical clinic Wednesday afternoon, October 6, 1909, in the Syms operating theatre of the hospital. The visitors were entertained by Dr. George E. Brewer, the attending surgeon, and his associate, Dr. Charles H. Peck. Their assistants were Dr. William E. Darrach and Dr. G. M. Phelps.

After an inspection of the hospital proper, escorted by Superintendent C. B. Grimshaw and the nurses, the guests were taken to the auditorium of the Syms theatre, where five operations were performed, covering a period of two hours. The first was the wiring of a fracture of the patella (knee cap); the second, an emergency case of appendicitis, the patient being a little boy named Frank Smith, brought to the hospital in an ambulance from his home. The third operation was for goitre; the fourth one for cancer of the breast, and the final one was a demonstration in nævus by Dr. Charles T. Dade—the removal of a birthmark by spraying with liquid air. All the operations were successful, and the visitors expressed themselves as surprised and pleased with the conditions found in the operating room, which, they said, impressed them as one of the best in the world, while the technic, they said, was as near perfect as they had ever seen.

